# Micro Fabry-Pérot Interferometer at Rayleigh Range

**DOI:** 10.1038/s41598-018-33665-8

**Published:** 2018-10-12

**Authors:** Yusuke Tsujiie, Yoshiyuki Kawamura

**Affiliations:** Department of Intelligent Mechanical Engineering, Faculty of Engineering, Fukuoka Institute of Technology, 3-30-1 Wajirohigashi, Higashiku, Fukuoka 811-0295 Japan

## Abstract

The Fabry-Pérot interferometer is used in a variety of high-precision optical interferometry applications, such as gravitational wave detection. It is also used in various types of laser resonators to act as a narrow band filter. In addition, ultra-compact Fabry-Pérot interferometers are used in the optical resonators of semiconductor lasers and fiber-optic systems. In this work, we developed a micro-scale Fabry-Pérot interferometer that was constructed within the Rayleigh range of the optical focusing system. The high precision that is conventionally required for the optical parallelism and the surface accuracy of the mirrors was not so critical for this type of Fabry-Pérot interferometer. The interferometer was constructed using a gold-coated silicon microcantilever with reflectivity of 92% and a dielectric multilayer flat mirror with reflectivity of 85%. The focal spot size of the laser beam is 20 μm and the cavity length is approximately 20 μm. The finesse was measured to be approximately 25. The interferometric characteristics of the device were consistent with the theoretically calculated performance. The developed micro Fabry-Pérot interferometer has the potential to make a marked contribution to advances in optical measurements in various micro sensing system.

## Introduction

In this work, we have developed a micro Fabry-Pérot (FP) interferometer with high sensitivity to realize high-performance feedback damping of the thermal vibration of a silicon microcantilever that is intended for use in an atomic force microscope (AFM). FP interferometers have been used in various high-precision optical interferometry applications, such as gravitational wave detection^1^. To date, there have been many studies of normal-sized FP interferometers^2,3^, but only a few studies have addressed smaller types of FP interferometers^4,5^.

There have been several studies of feedback cooling of the thermal vibration of micro cantilevers^6–13^. Recent studies found that the measured signal-to-noise ratio determines the limits of the feedback cooling performance^6,7,12^. We used an FP interferometer rather than a Michelson interferometer to improve the measurement sensitivity and thus increase the signal-to-noise ratio. In conventional FP interferometers, the polished end faces of optical fibers^10,11^ and micro mirrors from the surface of a multilayer dielectric mirror^12,13^ formed by focused ion beam microfabrication have been used as cavity mirrors. However, use of these methods for the mirror has led to issues such as low finesse due to optical diffraction from the fiber output aperture and problems with the parallelism of the optical alignment and the interferometer, along with difficulties in the microfabrication process. In addition, these methods do not use the merits of the Rayleigh range. In this work, we have developed a micro FP interferometer that uses the optical merits of the Rayleigh range of the focal system to simplify the optical system and improve the interferometric performance. The interferometric characteristics of this FP interferometer show good agreement with the theoretically calculated performance.

## Methods

**Figure 1:**
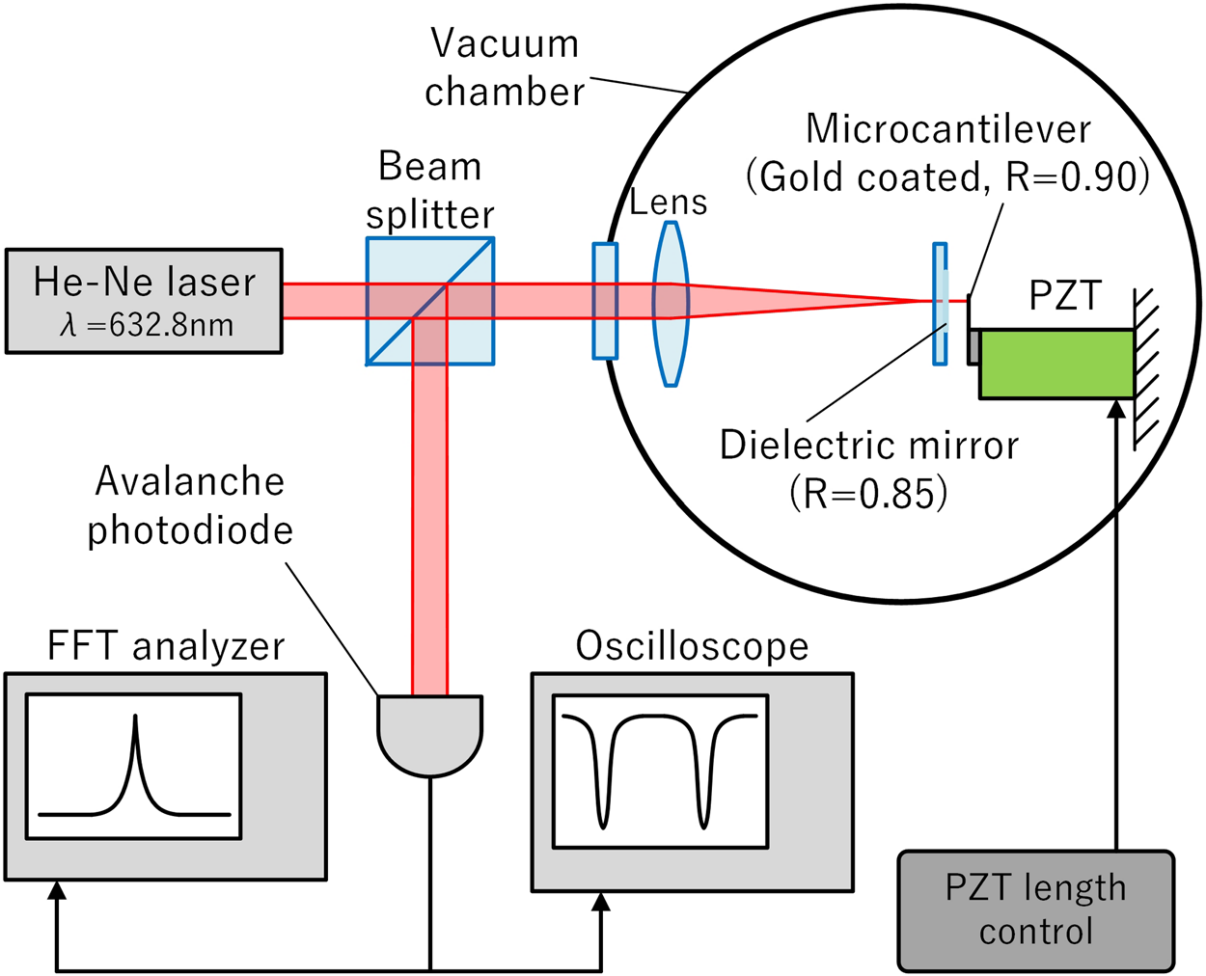
Micro Fabry-Pérot interferometer for vibration measurement system. The PZT is a piezoelectric actuator, and the FFT analyzer is a fast Fourier transform analyzer.

Figure 1 shows the experimental system that was used to measure the interferometric characteristics of the micro FP interferometer. A He-Ne laser (wavelength of 632.8 nm; laser power of approximately 1 mW) was used as the light source for the interferometer. The micro FP interferometer is constructed using the gold-coated surface of a microcantilever and a dielectric multilayer flat mirror. We measured the vibration of a commercially available silicon microcantilever (OMCL-AC240TN, Olympus Corporation) that is intended for use in AFMs.

**Figure 2:**
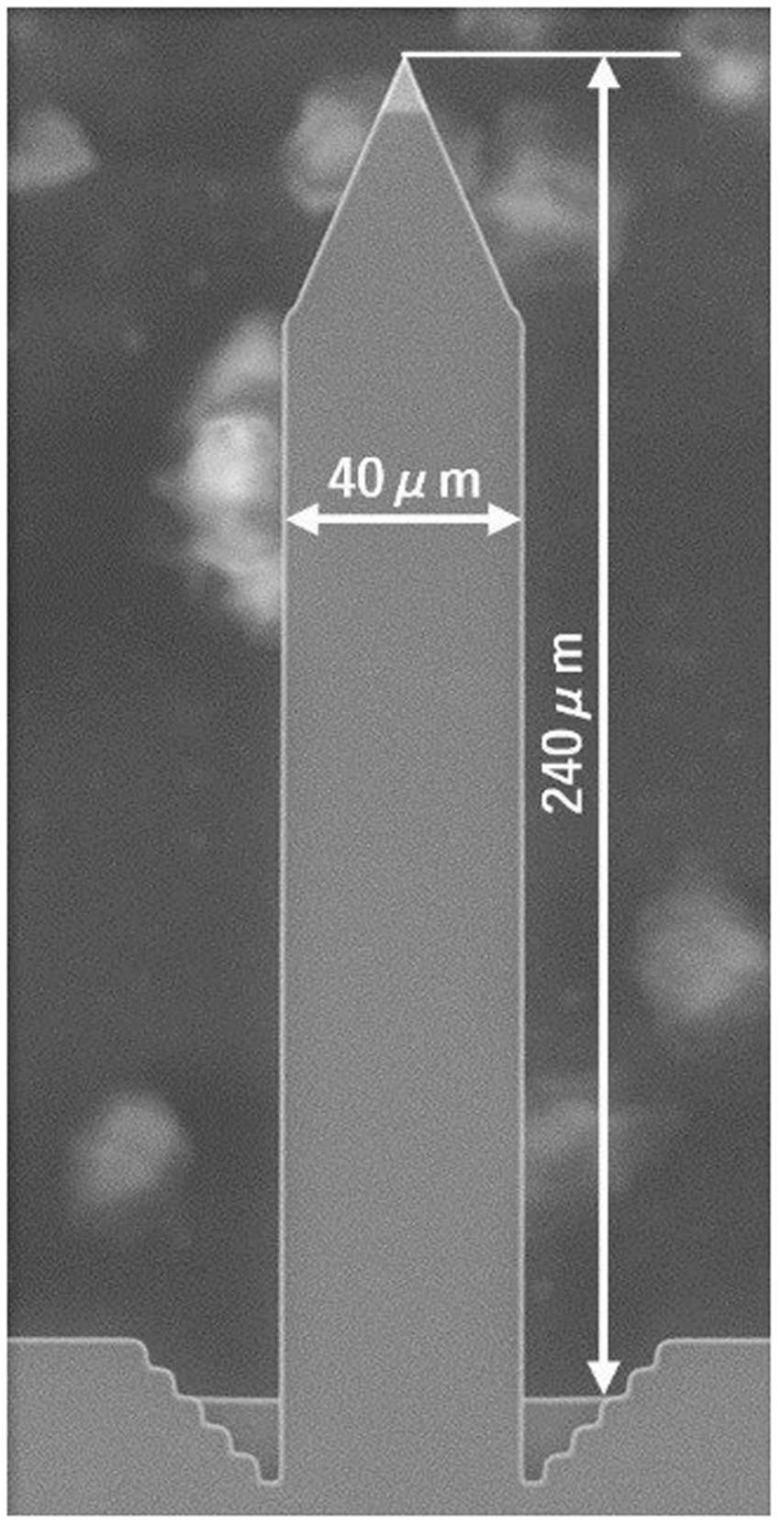
Scanning electron microscope image of the microcantilever.

Figure 2 shows a scanning electron microscope image of this microcantilever. The micro cantilever’s length, width, and thickness are 240 μm, 40 μm, and approximately 2.3 μm, respectively, and it is composed of single-crystal silicon. The natural oscillation frequency of the microcantilever is 77.6 kHz, and the catalog value of its spring constant is about 2 N/m. One single-side surface of the microcantilever was coated with gold to increase the laser reflectance using an ion-beam sputtering device that is commonly used for preprocessing before scanning electron microscope observation. The coating thickness was chosen to be as thin as possible while ensuring that sufficient reflectivity (92%) was obtained because reductions in both the natural oscillation frequency and the Q factor of the microcantilever were observed when a thick gold coating was used. The coating thickness was estimated to be approximately 25 nm based on the coating characteristic curve of the ion sputtering device. Ideally, the coating should be done only on the area, where the laser beam was irradiated. However the coating was done on the whole front surface of the cantilever, because it was easier than the partial coating. In case of the silicon micro cantilevers, we found dielectric multilayer coating was difficult on them. We tried several times, however the microlever were broken in all cases, probably due to the surface stress induced by the coating. It was one of the reason why we chose the gold coating.

The other side of the FP interferometer is formed by the dielectric multilayer flat mirror. The optical flatness and reflectance of this mirror were λ/10 and 85%, respectively. The diameter and thickness of the mirror were 30 mm and 1 mm, respectively.

A laser beam with a diameter of 4 mm was focused using a spherical lens, which has a focal length of 80 mm and an F number of 20. The focal spot size was estimated to be approximately 16 μm under the assumption of the diffraction limit. The Rayleigh range was estimated to be approximately 250 μm, and the cavity length was approximately 20 μm.

The optical system was set in a vacuum chamber at a pressure of approximately 4 × 10^−3^ Pa. The interferometric signal was separated using a beam splitter and measured using an avalanche photodiode. The microcantilever was driven using a lead zirconate titanate (PZT) piezoelectric actuator. The signal was measured using an oscilloscope and a fast Fourier transform (FFT) analyzer. The vacuum circumstance was not essential for this experiment. It was only for obtaining a clear thermal vibration signal of the micro cantilever. The same optical characteristics of it were also obtained in the atmospheric pressure.

## Results

The reflectance values of the microcantilever and the dielectric multilayer mirror were 92% and 85%, respectively. The reflectance of the microcantilever differs from that of the dielectric mirror. For the FP interferometer that was constructed using a pair of mirrors with different reflectances, the theoretical interferometric reflectance *R* was calculated to be

1 $$R=\frac{{R}_{1}+{R}_{2}-2\sqrt{{R}_{1}{R}_{2}}+4\sqrt{{R}_{1}{R}_{2}}{\sin }^{2}(\delta /2)}{1+{R}_{1}{R}_{2}-2\sqrt{{R}_{2}{R}_{2}}+4\sqrt{{R}_{1}{R}_{2}}{\sin }^{2}(\delta /2)},$$

where *R*_1_ and *R*_2_ are the reflectances of the multilayer mirror and of the microcantilever, respectively^14^. *δ* is the phase shift of each transmitted light wave due to the change in the cavity length *L*_*C*_ and is given by $\delta =4{\rm{\pi }}{L}_{{\rm{C}}}/\lambda$.

**Figure 3:**
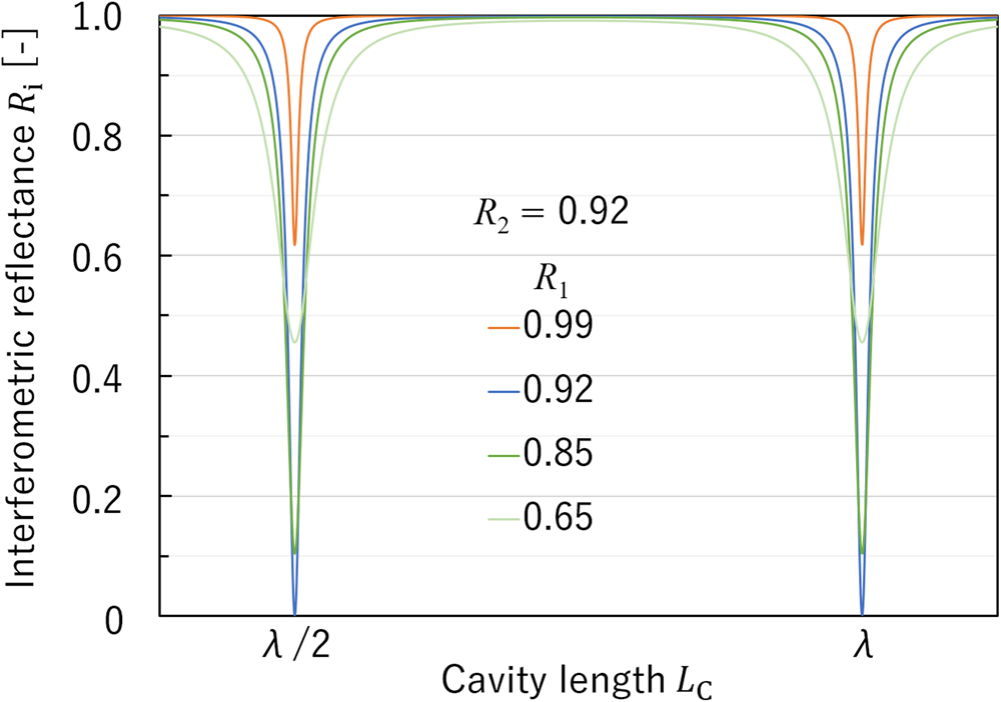
Interferometric reflectance ***R*** as a function of cavity length, calculated using eq. (2), for various values of ***R***_***2***_.

Figure 3 shows the interferometric reflectance *R* as a function of the cavity length, as calculated using eq. (1) for various values of *R*_2_. We can see that the minimum interferometric reflectance could not be 0% when *R*_1_ and *R*_2_ differ from each other. In the case where *R*_1_ = *R*_2_, the minimum reflectance is 0%. In the case where *R*_1_ > *R*_2_, the minimum reflectance increases as *R*_1_ decreases.

**Figure 4:**
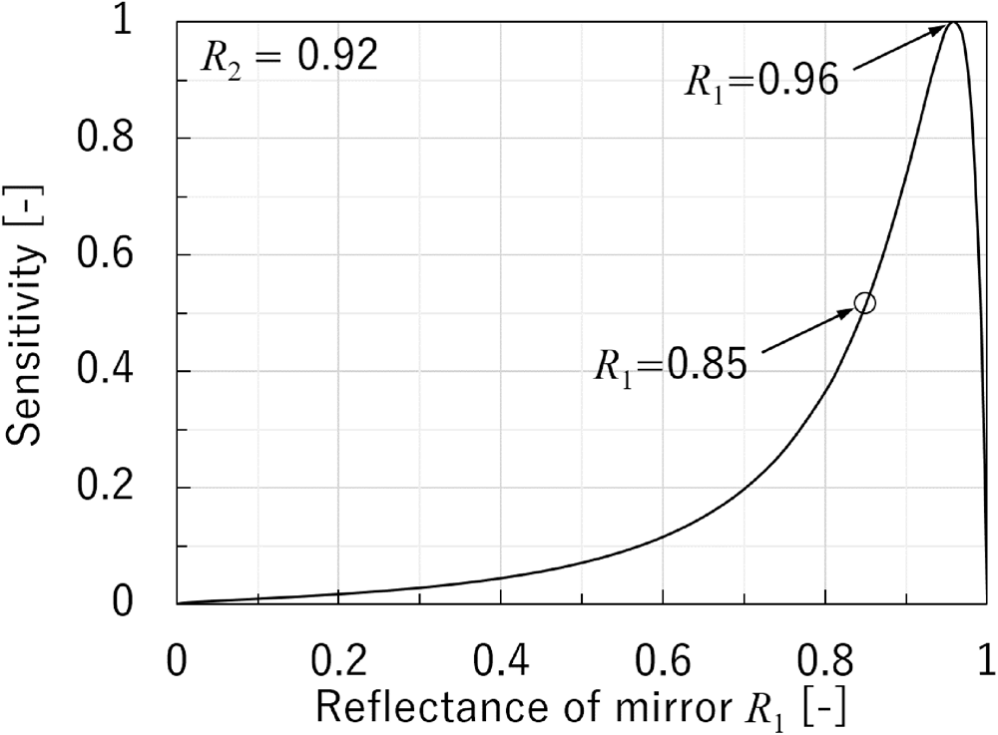
Calculated sensitivity for optimum FP design. The open circle represents the calculation for *R*_1_ = 0.85.

Figure 4 shows the relative maximum sensitivity of the FP interferometer as a function of *R*_1_ when *R*_2_ = 0.92, where the slope of the interferometric curve is maximized. The sensitivity reached a maximum value at *R*_1_ = 0.97 (i.e., not at *R*_1_ = 0.92). For the various values of *R*_2_, the maximum value of the sensitivity for *R*_2_ differed from that for *R*_1_ and was located between *R*_2_ and 1. The open circle in Fig. 4 is related to the experimental conditions (where *R*_2_ = 0.85).

**Figure 5:**
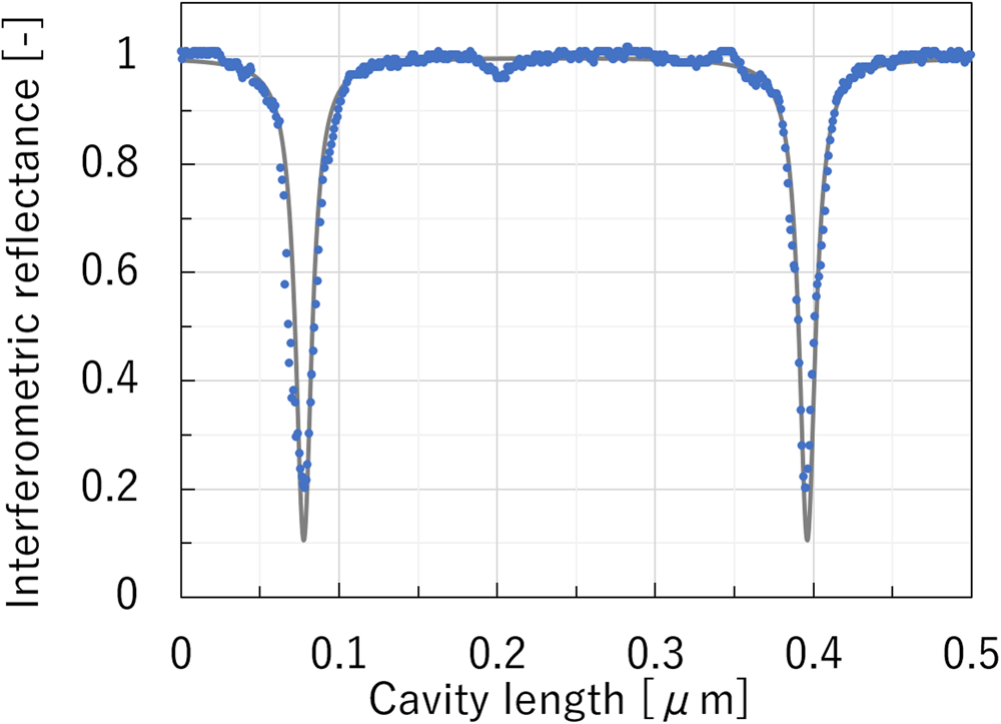
FP interferometric characteristics of the micro FP interferometer. The gray solid line shows the curve that was calculated theoretically using eq. (2). *R*_1_ and *R*_2_ are 0.85 and 0.92, respectively.

Figure 5 shows the reflectance of the micro FP interferometer as a function of cavity length. *R*_1_ and *R*_2_ were 0.85 and 0.92, respectively. The FP interferometric characteristics were measured by varying the cavity length using the PZT actuator. The gray solid line indicates the theoretical calculation results obtained using eq. (1). The blue solid circles are the experimental results, which showed good agreement with the values on the theoretically calculated curve. Scale fitting was only performed for the horizontal scale. The finesse of the interferometer was measured to be 25.

**Figure 6:**
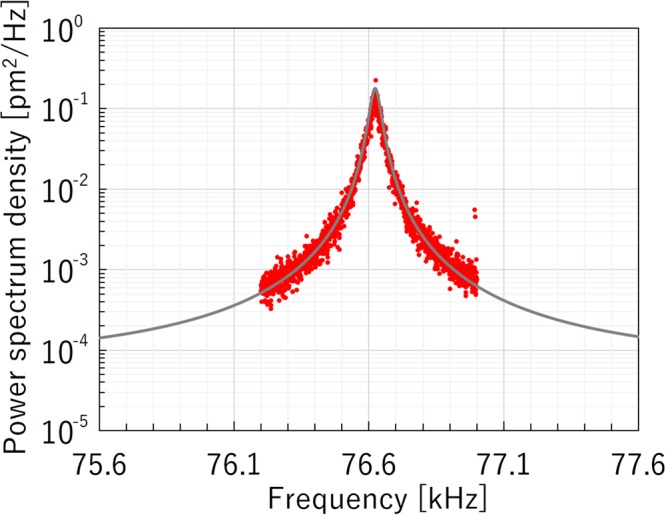
Power spectral density of the thermal vibration of the microcantilever. The solid line curve was fitted using a Lorentzian function.

Figure 6 shows the FFT signal of the thermal vibration of the microcantilever, which is used as one of the mirrors of the micro FP interferometer, at maximum sensitivity. The frequency resolution of the FFT analyzer is 0.5 Hz. The data are averaged over 1000 measurements. The gray solid line is fitted to the experimental results using a Lorentzian curve. The quality factor *Q* was measured to be approximately 2000. The thermal vibration amplitude was approximately 5 pm.

## Discussions

In the vicinity of the focal point of the focusing optical system, the laser beam wave fronts are sufficiently flat to allow the FP interferometer to be constructed.

The Rayleigh length *l*_*L*_ is given by

2 $${l}_{{\rm{L}}}\cong \lambda {(\frac{f}{D})}^{2},$$

where *λ* is the wavelength of the laser, *f* is the focal length and *D* is laser beam spot diameter on the lens. In this experiment, it was estimated to be about 250 μm, which is long enough than the cavity length (20 μm). It is the reason why flat mirrors can be used as the reflectors of the FP interferometer placed in the focusing optical system.

**Figure 7:**
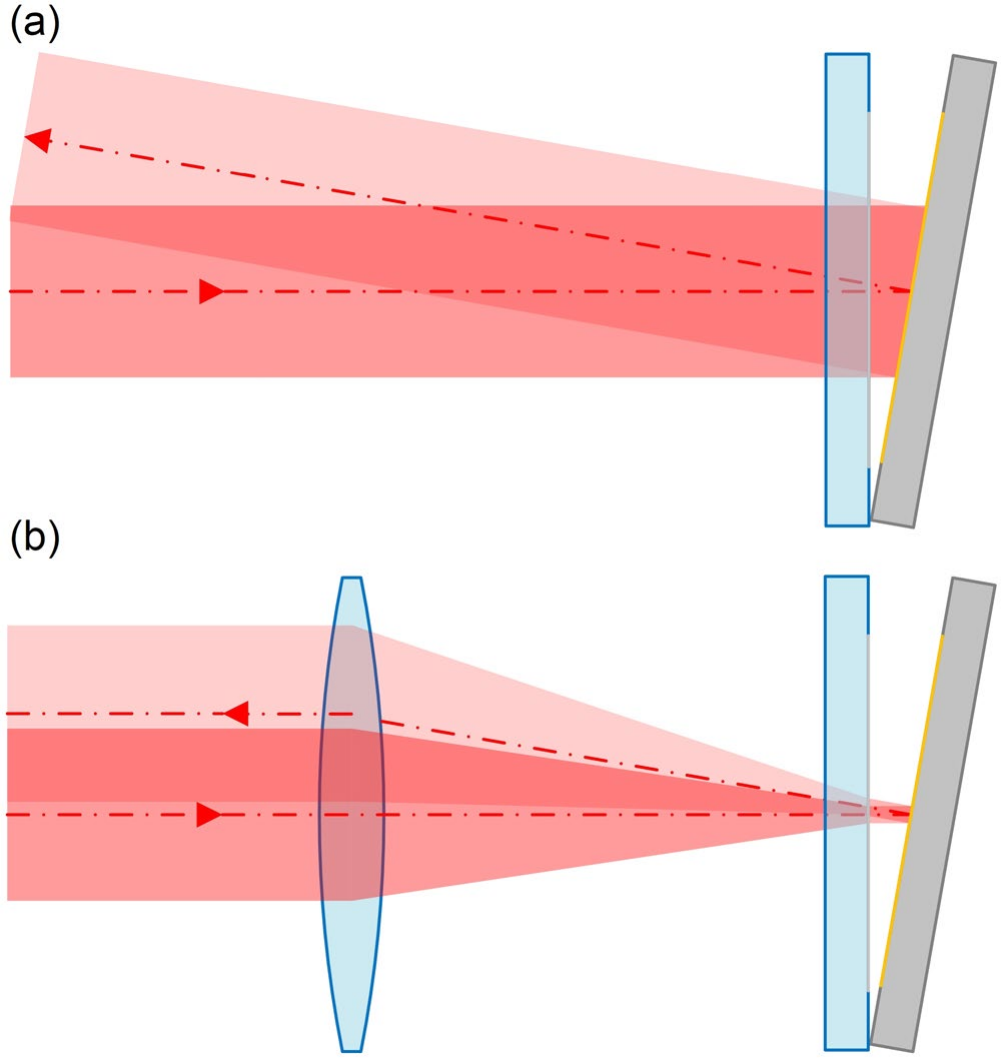
Retroreflectivity comparison of the two types of optical reflecting system. (**a**) Standard FP interferometer. (**b**) Micro FP interferometer.

Figure 7 shows a comparison of the retro reflectivity properties of the two types of optical reflecting systems, when the mirrors of the FP interferometer are not located in parallel with each other; this behavior is caused by the retroreflective effect. In case (b), the optical axis of the reflected beam is oriented parallel to the optical axis of the incident beam by the retroreflective effect, which makes it possible for the two beams to interfere. Consequently, in the micro FP interferometer, the requirement for parallel orientation of a pair of mirrors is greatly reduced when compared with the normal-type FP interferometer ((a)). We could observe the interference fringes, even when the reflected laser beam pattern from the interferometer was not completely overlapped with that of incident laser beam.

The demand for optical flatness in the micro FP interferometer is also much weaker when compared with that for the normal-type FP interferometer because of the reduced cross-sectional area of the laser beam. The optical flatness of the reflection mirror were only λ/10, by which we cannot obtained the fines of 25 in case of the normal type FP interferometer.

Another characteristic of the micro FP interferometer is that it has a large free spectral range because of its short cavity length.

For the practical fabrication of this type of FP interferometer, we think that it is one of the method to contact a small dielectric multilayer mirror to the basement of the microcantilever with a thin spacer using the optical contact bonding.

## Conclusions

We have developed a micro Fabry-Pérot interferometer that is constructed within the Rayleigh range of the optical focusing system and demonstrated that the interferometric characteristics of this interferometer were consistent with the theoretically calculated characteristics. The conventional high precision required for the optical parallelism and the surface flatness of the mirrors was not so essential for the micro FP interferometer. We believe that the proposed micro FP interferometer has the potential to make a marked contribution to advances in optical measurements in various micro sensing system.

## Data Availability

The authors declare that all the relevant data supporting this finding of this study are available within the article or from the corresponding author upon request.
